# Working life expectancy and working years lost among users of part- and full-time sickness absence in Finland

**DOI:** 10.5271/sjweh.4054

**Published:** 2022-12-30

**Authors:** Elli Hartikainen, Svetlana Solovieva, Eira Viikari-Juntura, Taina Leinonen

**Affiliations:** 1 The Finnish Institute of Occupational Health, TYÖTERVEYSLAITOS, Finland

**Keywords:** disability retirement, graded return to work, partial sick leave, prolonging working life, propensity score, quasi-experiment, return to work, Sullivan method, unemployment, work participation

## Abstract

**Objectives:**

The use of part-time sickness absence (pSA) instead of full-time sickness absence (fSA) is known to increase work participation. Yet, its effect on the total length of working lives remains unclear. We carried out a quasi-experiment to assess the impact of using pSA versus fSA on the length of working lives.

**Methods:**

We used a register-based 70% random sample of the working-age population living in Finland on 31 December 2007 to (i) form propensity-score-matched groups of users of pSA and fSA and (ii) calculate their working life expectancy (WLE) and working years lost (WYL). We applied the Sullivan method based on daily measured time spent at work and other labor market statuses, followed up over a four-year period until the end of year 2017. The study population consisted of private and public sector employees with SA due to mental and musculoskeletal disorders, ie, the diagnostic groups where pSA has been primarily used.

**Results:**

Among both genders, the pSA group had a significantly higher WLE at age 30 than the fSA group, with larger differences seen in mental disorders compared to musculoskeletal diseases and in the private versus public sector. Overall, the pSA group had fewer WYL due to unemployment and disability retirement but more expected years working with partial disability benefits than the fSA group.

**Conclusions:**

Based on beneficial working career effects, the use of pSA instead of fSA should always be recommended for persons with mental or musculoskeletal disorders where feasible.

Increasing work participation and extending working lives have become highly prioritized on the political agenda in many Western countries due to demographic ageing and related economic pressures. Making use of partial working capacity has been seen as an important tool for increasing work participation (1, 2). During the last decades, the Nordic countries as well as some countries in continental Europe have strongly begun to develop work disability policies to promote part-time work during sickness absence (SA) (3, 4).

In Finland, SA of permanent residents is compensated by the Social Insurance Institution of Finland after a waiting period of ten weekdays (including Saturday) that is typically paid by the employer (5). After the waiting period, part-time SA (pSA) is a voluntary option for persons who are eligible for full-time SA (fSA). Based on medical assessment, these individuals can work without harm to their health and part-time work can be arranged by their employer. pSA has been developed to help persons with reduced work ability to remain in work at least part-time and to return to work full-time. The partial sickness allowance is 50% of full allowance, and the employee works 40–60% of the time while receiving it. Full sickness allowance is obtainable for a maximum of 300 weekdays and partial sickness allowance for an additional 72 (at the time of the study, currently 120) weekdays accumulated over a two-year period. In the case of continuing work disability, a partial or a full disability pension can be granted.

Most previous studies from Finland, Norway, Germany and Canada suggest that pSA or graded return to work instead of fSA reduces the duration of SA, enhances return to work, and increases overall work participation (6–11), although a few studies from Denmark and The Netherlands have shown no such effects among employees with mental (12) or musculoskeletal (13) disorders. Along with the above-mentioned positive effects, studies have shown that pSA increases the likelihood of partial disability retirement, ie, the users of pSA transit to a more permanent partial work disability path (9, 14). Since the majority of partial disability pensioners work part-time (15), this path may help in maintaining attachment to working life and continuing to work at least part-time also in the long-term. However, partial disability retirement does not necessarily lengthen working lives if it reduces the attempts to return to full-time work. For these reasons, the effect of the use of pSA instead of fSA on the total length of working lives remains unclear.

The aim of the present study was to carry out a quasi-experiment to assess the impact of the use of pSA – rather than fSA – on the length of working lives. To this end, we used Finnish register data to (i) form propensity-score-matched groups of users of pSA and fSA and (ii) calculate their working life expectancy (WLE) and working years lost (WYL) due to different reasons. We applied the Sullivan method based on the proportion of time spent at work and other labor market statuses followed up over a four-year period. We restricted our study population to private and public sector employees with SA due to mental disorders and musculoskeletal diseases, ie, the diagnostic groups where pSA has been primarily used.

## Methods

### Data sources

The study base consisted of a 70% random sample of the working-age population living in Finland on 31 December 2007. Register-based longitudinal data were available for this sample until 31 December 2017.

The data included information on (i) episodes of employment, unemployment, earnings-related retirement, and vocational rehabilitation from the Finnish Centre for Pensions, (ii) episodes of compensated SA and national pensions obtained from the Finnish Social Insurance Institution, and (iii) demographic factors, education, occupation, industrial sector and income obtained from the FOLK data of Statistics Finland. Data from these three registers were linked on the basis of social security numbers of the participants, pseudonymized for analyses.

Work exposures, including physical heaviness of work and job control, were estimated by linking information from gender-specific job exposure matrices (JEM) to occupational titles in the register data. The JEM were developed earlier in a large population survey- and interview-study and have been described in more detail elsewhere (16, 17).

### Study design

In this quasi-experimental study, we compared WLE and WYL between those who chose to take pSA instead of fSA and those who used only fSA. Even though in Finland pSA is available immediately after the waiting period, the vast majority of pSA users have a preceding fSA period (2, 9). We therefore investigated those who, after an initial period of fSA, either switched to pSA or had a new fSA period. The time point of deciding on the continuation of the SA, as either part- or full-time, was considered as the point of “random assignment” to the users of pSA or fSA. We followed the same approach taken in a previous Finnish study using a similar matching design (9).

For this purpose, we included 30–62 year-old individuals who had an onset of a pSA or fSA spell between 1 January 2010 and 31 December 2013 (hereafter called index spells) and had a preceding fSA spell that ended 1–31 days before the start of the index spell (resulting in fSA–pSA and fSA–fSA sequences). The pSA and fSA index spells were derived by first identifying all eligible fSA–pSA and fSA–fSA sequences for an individual within each of the four calendar years. A calendar year was chosen to be able to perform matching within the different yearly strata, which is more specifically described below. Within a calendar year, we then gave priority to the fSA–pSA sequences to capture all individuals eligible for the pSA group. Among multiple sequences of the same type, we chose the one occurring first during the year. This resulted originally in 17 608 fSA–pSA and 110 468 fSA–fSA sequences.

We excluded persons who were not employed in the private or public sector at the start of the index spell (1096 fSA–pSA and 60 918 fSA–fSA sequences). We also excluded persons who had accumulated >175 calendar days (corresponding to 150 compensated days, including weekdays and Saturdays) of fSA (3761 fSA–pSA and 11 157 fSA–fSA sequences) or >42 calendar days (corresponding to 36 compensated days) of pSA (169 fSA–pSA and 314 fSA–fSA sequences) during the preceding two years before the index spell. These limits were agreed upon to exclude persons who would be close to the maximum limit of compensated days, which in Finland was 300 days for fSA and 72 days for pSA at the time of the study. Finally, we excluded persons whose index spell was due to causes other than musculoskeletal diseases (M00-M99 according to ICD-10) or mental disorders (F00-F99) (3066 fSA–pSA and 17 345 fSA–fSA sequences).

The final pools eligible for the pSA and fSA groups consisted of 9516 and 20 734 sequences, respectively. The four-year follow-up started on the last day of the index spell.

### Working life expectancy and working years lost

For the calculations of WLE (sum of the time expected to be spent at work, in this case full work duties) and WYL (sum of the expected working time lost), information on age-specific labor market participation was derived based on the proportions of the four-year follow-up time spent in seven daily measured statuses (i): work (having employment and not receiving work disability, unemployment or pension benefits) (ii), partial work disability (receiving a partial work disability benefit, including pSA or partial disability retirement while having employment) (iii), time-restricted work disability (receiving a full-time work disability benefit paid for a restricted time period, including fSA, temporary disability retirement or vocational rehabilitation) (iv), unemployment (v), other non-employment (receiving other benefits, no benefits or being an emigrant (vi), disability retirement (full disability retirement irrespective of having employment or partial disability retirement without employment), and (vii) other permanent retirement (non-disability retirement).

### Statistical methods

*Propensity score matching*. Propensity score matching was applied to control for the confounding effect of observed background factors, ie, sociodemographic and work-related factors as well as labor market history, on the difference in WLE and WYL between the pSA and fSA groups. The maximum number of matched pairs was determined by the size of the pool eligible for the pSA group, ie, 9516 index spells.

Recommendations from systematic reviews for modeling propensity scores and applying propensity score matching were followed (18, 19). Prior to the propensity score calculation, differences in the distributions of background variables between the pSA and fSA pools were examined. No outliers in the background variables were found in either the pSA or fSA pool.

To calculate the propensity score in each exactly matched strata (see below), a set of hierarchical logistic regressions was conducted with a set of background factors as covariates and having pSA as the index spell (belonging to the pSA pool) as the dependent variable. Covariates used for controlling sociodemographic and work-related factors in the propensity score models were age (continuous variable), region of residence (Southern, Western, Eastern, and Northern Finland), industrial sector (dummy variables), physical heaviness of work (proportion exposed), job control (mean score) and total earned income (continuous variable) in the calendar year prior to that of the onset of the index spell, whereas labor market history was controlled by using the number of preceding pSA, fSA, unemployment and employment days (continuous variables) and having temporary disability retirement or vocational rehabilitation (yes/no) during two preceding years.

Matching was performed in different stratas by calendar year (2010, 2011, 2012, 2013), gender (men, women), age group (30-44, 45-62), disease group of SA (mental disorders, musculoskeletal diseases), employment sector (private, public) and education (primary, secondary, tertiary). Five strata did not exist in the pSA pool. Of the total 192 (4×2×2×2×2×3) possible strata, matching was done in the remaining 187 strata. For 11 strata, the pSA pool was larger than the fSA pool, all of them, including employees of the private sector, with tertiary education. In order to maximize the number of matched pairs, the matching was done in two steps (i): matching within the original 187 strata and (ii) from the remaining pool of pSA and fSA, matching within strata by calendar year, gender, age group, disease group and employment sector, allowing for primary-secondary and secondary-tertiary education combinations.

After the first step 8213 (86.3%) matched pairs were formed. In the second step 347 additional matched pairs were found. In total, 8560 (90.0%) matched pairs were included into the further analyses.

Due to separate selection of index spells within each calendar year, the same individual may have contributed to the formed pools of pSA and fSA more than once. This was found to affect 660 matched pairs. The effects of multiple inclusions of the same individual on the estimates of WLE and WYL were addressed in a sensitivity analysis. For this sensitivity analysis, an individual who had multiple records contributing to both pSA and fSA groups was kept only in the pSA group. If the individual contributed more than once to the same group (either pSA or fSA), the record of the earliest calendar year was kept.

The SPSS v.27 statistical software (IBM, Armonk, NY, USA) was used for the propensity score analyses.

*Sullivan method*. We used the Sullivan method (20) adopted for the estimation of healthy life expectancy (21) to calculate years expected to be spent in different work participation statuses. We calculated working life tables by estimating the average probability of survival across years 2010–2013 in the general Finnish population between 30 to 62 years and used the proportion of time spent in different work participation statuses during the four-year follow up at single years of age as the entity for estimation of time expected to be spent at work (WLE) and in other work participation statuses (WYL).

The WLE and WYL with their 95% confidence intervals (CI) correcting for variation in mortality were calculated for the pSA and fSA groups according to guidelines provided by Jagger et al (2006). The estimates can be interpreted as the time that employees – who every fourth year during the remaining of their working careers always choose either pSA or fSA after a period of fSA– are expected to spend in different labor market statuses after a given age, assuming that they experience the same age-specific labor market participation and mortality rates that were observed during the study period. The approach used thus captures the effects of a repeated use of pSA instead of fSA on WLE and WYL.

## Results

After propensity score matching, the study population consisted of 2021 male and 6539 female pairs. The distributions of the background factors were balanced between the matched pSA and fSA groups (table 1).

**Table 1:** Distributions of the matched part-time (pSA) and full-time (fSA) sickness absence ) groups by background factors for men and women.

	Men				Women			
	
	pSA group		fSA group		pSA group		fSA group	
			
	N	% (Mean)	N	% (Mean)	N	% (Mean)	N	% (Mean)
Index year								
2010	323	16.0	323	16.0	1146	17.5	1146	17.5
2011	444	22.0	444	22.0	1467	22.4	1467	22.4
2012	540	26.7	540	26.7	1803	27.6	1803	27.6
2013	714	35.3	714	35.3	2123	32.5	2123	32.5
Age group (years)		(46.6)		(46.9)		(47.5)		(47.8)
30–44	795	39.3	795	39.3	2280	34.9	2280	34.9
45–62	1226	60.7	1226	60.7	4259	65.1	4259	65.1
Disease group								
Mental	747	37.0	747	37.0	2628	40.2	2628	40.2
Muscoloskeletal	1274	63.0	1274	63.0	3911	59.8	3911	59.8
Employment sector								
Private	1696	83.9	1696	83.9	3253	49.8	3253	49.8
Public	325	16.1	325	16.1	3286	50.2	3286	50.3
Education								
Primary	353	17.5	342	16.9	707	10.8	725	11.1
Secondary	1080	53.4	1108	54.8	3201	49.0	3213	49.1
Tertiary	588	29.1	571	28.3	2631	40.2	2601	39.8
Industrial sector ^a^								
Manufacturing	627	31.0	642	31.8	577	8.8	571	8.7
Construction	153	7.6	157	7.8	48	0.7	57	0.9
Wholesale & retail trade	182	9.0	193	9.6	653	10.0	647	9.9
Transportation & storage	245	12.1	245	12.1	207	3.2	203	3.1
Accomodation & food service activities	44	2.2	39	1.9	303	4.6	292	4.5
Knowledge work, administrative & support service activities, etc.	421	20.8	379	18.8	1506	23.0	1540	23.6
Education	72	3.6	75	3.7	359	5.5	333	5.1
Human health & social work activities	116	5.7	127	6.3	2548	39.0	2570	39.3
Arts, entertainment & other service activities	69	3.4	72	3.6	240	3.6	228	3.5
Other	61	3.0	63	3.1	53	0.8	62	1.00
Missing	31	1.5	29	1.4	45	0.7	36	0.5
Income (€/year)^b^								
≤30 000	489	24.2	530	26.3	3232	49.4	3510	53.7
30 001–60 000	1338	66.2	1288	63.7	3130	47.9	2772	42.4
>60 000	194	9.6	203	10.0	177	2.7	257	3.9
Region								
Southern	756	37.4	747	37.0	2400	36.7	2351	36.0
Western	468	23.2	462	22.9	1558	23.8	1593	24.4
Eastern	450	22.3	438	21.7	1310	20.0	1303	19.9
Northern	347	17.2	374	18.5	1271	19.4	1292	19.8
Physically heavy work ^b^								
<40% exposed	1163	57.6	1187	58.7	4547	69.5	4563	69.8
≥40% exposed	858	42.4	834	41.3	1992	30.5	1976	30.2
Job control score ^b^								
>median (high)	1264	62.5	1185	58.6	3173	48.5	3099	47.4
≤median (low)	757	37.5	836	41.4	3366	51.5	3440	52.6
Employment days ^b,c^								
<365	20	1.0	16	0.8	54	0.8	51	0.8
365–729	306	15.1	325	16.1	644	9.9	672	10.3
730	1695	83.9	1680	83.1	5841	89.3	5816	88.9
Unemployment days ^b,c^								
0	1783	88.2	1770	87.6	6106	93.4	6105	93.4
1–30	88	4.4	104	5.2	193	3.0	158	2.4
31–180	111	5.5	114	5.6	164	2.5	203	3.1
181–730	39	1.9	33	1.6	76	1.2	73	1.1
pSA days ^b,c^								
0	1999	98.9	2016	99.8	6451	98.7	6499	99.4
1–42	22	1.1	5	0.2	88	1.3	40	0.6
fSA days ^b,c^								
1–30	322	15.9	433	21.4	1109	16.9	1452	22.2
31–90	923	45.7	708	35.0	2979	45.6	2427	37.1
91–175	776	38.4	880	43.5	2451	37.5	2660	40.7
Temporary disability retirement ^c^								
No	1976	97.8	1980	97.97	6447	98.6	6469	98.9
Yes	45	2.2	41	2.03	92	1.4	70	1.1
Vocational rehabilitation ^c^								
No	2011	99.5	2014	99.65	6476	99.0	6478	99.1
Yes	10	0.5	7	0.35	63	1.0	61	0.9
Total	2021	100.0	2021	100.0	6539	100.0	6539	100.0

a Largest groups are shown separately.

b Income, physically heavy work, job control score, employment days, unemployment days, pSA days and fSA days have been categorized for descriptive purposes.

c During the preceding two years.

**Figure 1:**
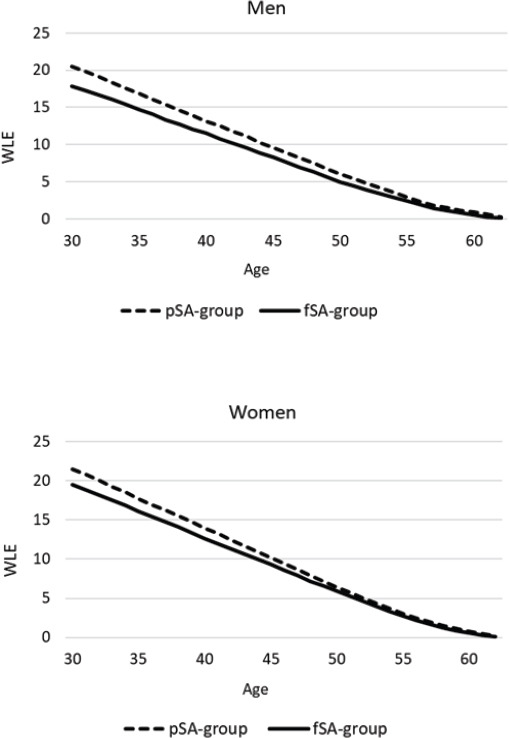
Working life expectancy (WLE, years) at age 30–62 among the matched part-time (pSA) and full-time (fSA) sickness absence groups by gender.

The pSA group had a higher WLE than the fSA group among both genders and at all ages (figure 1). At age 30, WLE was 20.51 years for men and 21.45 years for women in the pSA group and 17.88 years for men and 19.47 years for women in the fSA group (table 2). Men in the pSA group were therefore expected to work 2.63 years more than men in the fSA group, whereas the corresponding difference was 1.98 years among women. Furthermore, in both genders the pSA group was expected to have fewer WYL due to unemployment and disability retirement and more expected years working with partial work disability than the fSA group. The absolute differences between the groups were 1.18 for unemployment, 1.07 for disability retirement and 0.94 for partial work disability among men. Among women the corresponding differences were 1.14, 0.85 and 0.87 years, respectively. Additionally, the pSA group was expected to lose fewer years due to time-restricted work disability, other non-employment and other retirement, but the differences were smaller and mostly non-significant. Sensitivity analyses, which were performed by excluding multiple records of individuals, showed similar results as the main analyses (supplementary material www.sjweh.fi/article/4054, table S1).

**Table 2:** Working life expectancy (WLE) and working years lost (WYL) among the matched part-time (pSA) and full-time (fSA) sickness absence groups at age 30 by gender. [CI=confidence interval]

	pSA group	95% CI	fSA group	95% CI	Difference	95% CI
Men						
WLE	20.51	19.81–21.20	17.88	17.22–18.55	2.63	1.26–3.98
WYL						
Partial work disability	1.61	1.32–1.91	0.67	0.48–0.86	0.94	0.46–1.43
Time-restricted work disability	3.94	3.47–4.41	4.42	3.93–4.90	-0.48	-1.43–0.48
Unemployment	2.10	1.76–2.45	3.28	2.85–3.71	-1.18	-1.95– -0.40
Other non-employment	0.85	0.62–1.07	1.15	0.89–1.41	-0.30	-0.79–0.18
Disability retirement	0.87	0.67–1.08	1.94	1.65–2.23	-1.07	-1.56– -0.57
Other permanent retirement	1.88	1.47–2.29	2.43	2.16–2.69	-0.55	-1.22–0.13
Women						
WLE	21.45	21.06–21.84	19.47	19.09–19.85	1.98	1.21–2.75
WYL						
Partial work disability	1.93	1.75–2.11	1.06	0.93–1.19	0.87	0.56–1.18
Time-restricted work disability	4.35	4.07–4.63	4.64	4.35–4.92	-0.29	-0.85–0.28
Unemployment	1.48	1.31–1.66	2.62	2.39–2.84	-1.14	-1.53– -0.73
Other non-employment	0.74	0.61–0.87	1.04	0.89–1.19	-0.30	-0.58– -0.02
Disability retirement	0.52	0.43–0.62	1.37	1.24–1.51	-0.85	-1.08– -0.62
Other permanent retirement	1.95	1.73–2.16	2.22	2.08–2.36	-0.27	-0.63–0.08

Within the disease groups, the differences in WLE and WYL between the pSA and fSA groups were similar among men and women, whereas within the employment sectors the differences between the genders could not be evaluated due to small numbers of men in the public sector (supplementary figure S1). We pooled the genders for the further group-specific calculations. At age 30 years, the pSA group had a higher WLE than the fSA group in both mental disorders and musculoskeletal diseases (table 3) as well as in the private and public sector (table 4). Within the diagnostic group of mental disorders, the differences in WLE between the pSA and fSA groups were clearly larger (3.92 years) than within musculoskeletal diseases (1.02 years). Regarding WYL within mental disorders, the absolute differences between the pSA and fSA groups were largest for unemployment and disability retirement. Within musculoskeletal diseases, the pSA group spent one year longer than the fSA group working with partial work disability. The fSA group again spent more time in unemployment and on disability retirement than the pSA group, although the differences tended to be smaller than those in mental disorders. Furthermore, within the private sector, the differences in WLE in favor of the pSA group were larger (2.86 years) than within the public sector (1.03 years). The difference between the pSA and fSA groups in WYL due to unemployment appeared to be larger in the private than the public sector.

**Table 3:** Working life expectancy (WLE) and working years lost (WYL) among the matched part-time (pSA) and full-time (fSA) sickness absence groups at age 30 by main diagnostic group. [CI=confidence interval]

	pSA group	95% CI	fSA group	95% CI	Difference	95% CI
Mental disorders						
WLE	21.17	20.46–21.88	17.25	16.58–17.91	3.92	2.55–5.30
WYL						
Partial work disability	1.47	1.17–1.77	0.87	0.65–1.08	0.60	0.09–1.12
Time-restricted work disability	4.23	3.73–4.72	4.98	4.47–5.48	-0.75	-1.75–0.25
Unemployment	1.73	1.41–2.05	3.22	2.79–3.64	-1.49	-2.23– -0.79
Other non-employment	0.77	0.55–0.98	1.27	0.99–1.55	-0.50	-1.00– -0.01
Disability retirement	0.61	0.43–0.80	1.88	1.61–2.16	-1.27	-1.73– -0.81
Other permanent retirement	1.80	1.36–2.23	2.31	2.05–2.57	-0.51	-1.21–0.18
Musculoskeletal diseases						
WLE	20.99	20.60–21.37	19.97	19.60–20.34	1.02	0.26–1.77
WYL						
Partial work disability	1.96	1.78–2.15	0.97	0.84–1.09	0.99	0.69–1.31
Time-restricted work disability	4.20	3.93–4.48	4.24	3.97–4.52	-0.04	-0.59–0.51
Unemployment	1.50	1.32–1.68	2.37	2.16–2.59	-0.87	-1.27– -0.48
Other non-employment	0.70	0.57–0.83	0.87	0.74–1.01	-0.17	-0.44–0.09
Disability retirement	0.56	0.74–0.65	1.26	1.13–1.39	-0.70	-0.93– -0.48
Other permanent retirement	1.87	1.66–2.08	2.10	1.96–2.23	-0.23	-0.57–0.12

**Table 4:** Working life expectancy (WLE) and working years lost (WYL) among the matched part-time (pSA) and full-time (fSA) sickness absence groups at age 30 by employment sector. [CI=confidence interval]

	pSA group	95% CI	fSA group	95% CI	Difference	95% CI
Private sector						
WLE	20.63	19.95–21.32	17.77	17.10–18.43	2.86	1.52–4.22
WYL						
Partial work disability	1.45	1.16–1.73	0.70	0.51–0.90	0.75	0.26–1.22
Time-restricted work disability	3.91	3.44–4.38	4.46	3.98–4.95	-0.55	-1.51–0.4
Unemployment	2.24	1.88–2.61	3.71	3.26–4.16	-1.47	-2.28– -0.65
Other non-employment	1.03	0.76–1.31	1.35	1.07–1.64	-0.32	-0.88–0.24
Disability retirement	0.64	0.45–0.82	1.59	1.33–1.85	-0.95	-1.40– -0.51
Other permanent retirement	1.87	1.48–2.27	2.20	1.95–2.44	-0.33	-0.96–0.32
Public sector						
WLE	21.54	21.16–21.93	20.51	20.15–20.88	1.03	0.28–1.78
WYL						
Partial work disability	2.20	2.01–2.40	1.22	1.08–1.36	0.98	0.65–1.32
Time-restricted work disability	4.63	4.35–4.92	4.64	4.35–4.93	-0.01	-0.58–0.57
Unemployment	0.70	0.58–0.83	1.34	1.17–1.51	-0.64	-0.93– -0.34
Other non-employment	0.33	0.25–0.42	0.60	0.49–0.72	-0.27	-0.47– -0.07
Disability retirement	0.53	0.43–0.63	1.30	1.17–1.43	-0.77	-1.00 – -0.54
Other permanent retirement	1.83	1.60–2.05	2.15	2.00–2.30	-0.32	-0.70–0.05

## Discussion

We used nationwide register-based data to assess the impact of using pSA rather than fSA on WLE and WYL in the Finnish employed population. We found that at all ages between 30 and 62 years, the use of pSA showed positive effects on the expected remaining length of working lives.

Our results indicated that at the age of 30 years, repeated use of pSA instead of fSA during one’s remaining working career would increase the expected time spent at work by around two years. A difference was found in both mental disorders and musculoskeletal diseases and within the private and public sector, the expected gain being greatest among persons with mental disorders and private sector employees. Overall, the favorable effects of taking pSA were attributable to shortened unemployment and disability retirement time. Also, the expected time working while receiving partial work disability benefits was 7–12 months longer among users of pSA compared to fSA.

In this study, we examined the effects of using pSA rather than fSA on the total expected remaining working careers of individuals. As far as we are aware, this kind of study has not been conducted earlier, although several studies have examined the effects of pSA on work participation in the shorter term. Our results are in line with previous studies, suggesting that the use of pSA or graded return to work has positive effects on work participation (6–11). The novel findings of the current study on the users of pSA and their matched controls consisting of fSA users suggest pSA to be an effective way to increase time spent at work during the remaining working careers among persons with mental or musculoskeletal problems.

The beneficial working-career effects of the use of pSA as opposed to fSA can be understood with the fact that persons who return to part-time work will adopt their daily routines and social contacts at the workplace. Even though the evidence of the effect of work on health is somewhat limited, work participation has been reported to have several beneficial effects on mental (22) and physical health (23) as well as general wellbeing (24). Furthermore, part-time working during disability has been reported to have positive effects especially on subjective health ratings among employees with musculoskeletal diseases (25) and mental disorders (26). Therefore, at least if managed well and supported with adequate resources, attending work during disability has the potential to benefit health and performance among employees suffering from mental or musculoskeletal problems. Furthermore, in cases when return to full work duties is not possible, the use of pSA often initiates a more long-term partial work disability path, during which the individuals concerned continue to participate partially in the labor market. A pathway from pSA to partial disability retirement has been observed also in previous studies (9, 14).

In this study, the largest gain in expected remaining work time at age 30 – around four years – was found for those who would repeatedly use pSA due to mental disorders. This was mostly attributable to reduced time spent in unemployment and disability retirement. It appears that for persons with mental health problems, pSA enhances recovery and hastens return to full duties. However, it is likely that those with the most severe mental disorders did not end up in our study population of employed persons, which may partly explain the very favorable results. Previous studies have shown mixed results regarding the effectiveness of pSA or graded return to work on work participation among employees with mental problems. Some have shown positive effects (7, 9, 10, 26) and others no effect (12) or effects only after longer-term fSA (27, 28). These mixed results may, at least to some extent, be explained by the differences in the social insurance systems, country-specific decision processes, involved stakeholders and other factors such as prescribing physician or patient selection mechanisms affecting whether or not pSA is preferable to fSA. Also the differences in the time point of starting part-time work in the rehabilitation process as well as differences in combining pSA with a clinical intervention could explain these mixed results.

Our findings indicated that, for those who would repeated take pSA due to musculoskeletal diseases, the expected gain in remaining work time at age 30 was only around one year. Return to full duties with musculoskeletal problems thus appears to be difficult even after a period of gradual part-time return to work. Nevertheless, pSA due to musculoskeletal diseases further increased the expected partial work participation with partial work disability benefits by an additional year. Moreover, although the gain in working years was more modest among those with musculoskeletal compared to mental disorders, the large size of the group of persons with SA due to musculoskeletal diseases adds to the significance of this gain at the population level.

The larger gain in the expected work time for pSA in the private versus public sector appears to indicate that pSA hastens return to full duties particularly in the private sector. This may relate to less stable working careers in the private sector leading to a larger advantage of initially returning to work at least part time. This is supported by the finding that the use of pSA appeared to reduce the time spent in unemployment to a larger extent in the private versus public sector.

The strengths of our study include the nationally representative register data, which have enough statistical power to study specific SA groups and do not have the problem of non-response or loss to follow-up. It is also noticeable that pSA users were matched with fSA users using rich information on sociodemographic factors and labor market history, which made the groups well comparable to study the effectiveness of pSA. Furthermore, as pSA is a voluntary arrangement in Finland for persons who have been medically assessed to be unable to work in their current work, it was possible to select a concurrent comparison group from those who did not choose this option, ie, the fSA group.

It should be kept in mind that WLE estimates are prognostic in nature and therefore based on the assumption that the age-specific behavioral patterns at the time of the study remain the same in the future. This holds only if the underlying conditions, such as the economic situation, are relatively stable. In this study, WLE and WYL estimations were based on labor market participation during follow-up periods of four years among a hypothetical group of employees, who would have always chosen either pSA or fSA after a period of fSA throughout their remaining working career. In reality, people may have just one such sequence, or they may successively move between the different types of sequences. However, having multiple pSA events was relatively common even during the four-year follow-up time of the study subjects. Furthermore, the use of pSA has been increasing in Finland (29).

The four-year follow-up time enabled us to capture long-term patterns in labor market participation after the use of pSA and fSA. We also had detailed information on various labor market statuses. However, many of the states that we were interested in were relatively rare, therefore resulting in a low number of transitions or no transitions between some of the states. We thus chose the Sullivan method, allowing us to examine the proportions of time spent in a larger number of different labor market statuses, which we would not have been able to do with methods based on transitional approaches, ie, multistate models.

A possible limitation of the current study is that we may not have been able to fully account for all relevant factors through the used matching procedure, ie, the pSA group may have been selected according to factors that could not be observed in the used register data, leading to a bias in our results. Such factors may include, eg, persons’ working motivation, health conditions other than those captured by preceding work disability, or more specific work environment factors. However, a study by Caliendo et al (30) showed that if detailed labor market histories of the individual were included in the matching procedure, characteristics such as personality traits hardly changed the estimated treatment effects of active labor market programmes even though they played a significant role in selection into the programmes. Therefore personal characteristics, especially those that are constant over time, appear to be well captured by prior labor market performance. The findings are likely to apply to other employment-enhancing interventions such as pSA. Hence, it is unlikely that any remaining variation in selection into the different types of SA – driven by unobserved personality traits – largely affected our results.

Potential unobserved confounding may nevertheless have arisen from the use of pSA indicating a more stable work situation as it requires that the employer arranges part-time work while the partial sickness allowance is paid. It is therefore possible that the shorter expected time on unemployment among the pSA group compared to the fSA group is partly explained by differences in working history and previous working life attachment, which we may not have been able to fully control for, even though we used unemployment days and other labor market history for matching. Furthermore, since the strata used were not based on specific diagnoses but on main diagnostic groups only, a bias could have arisen if those continuing on fSA had more severe diseases. However, this bias is unlikely to have largely influenced our findings, as we used information on previous SA days and other work disability history for matching. Furthermore, our study population consisted of employed individuals with a relatively short SA history, indicating diseases that are at an early stage.

The findings of the present study apply only to individuals with SA due to mental or musculoskeletal disorders. It is possible that the effectiveness of pSA differs for other disease groups. Furthermore, the generalizability of the results is restricted to countries with social security systems similar to that of Finland.

### Concluding remarks

Using pSA rather than fSA leads to longer working lives due to less time spent especially in unemployment and disability retirement. Particularly for persons with mental health problems or working in the private sector, pSA appears to increase the working years by enhancing return to full duties. For persons with musculoskeletal diseases, the gain in working years is more modest, yet at the population level still considerable as musculoskeletal diseases are one of the leading causes of work disability. Overall, using pSA instead of fSA leads to a notable increase in work time while receiving partial work disability benefits. The use of pSA instead of fSA should always be recommended for persons with mental health or musculoskeletal disorders where feasible.

## Supplementary material

Supplementary material

## References

[1] OECD How Good is Part-Time Work? (2010). In:OECD Employment Outlook 2010:Moving beyond the Jobs Crisis.

[2] Leinonen T, Solovieva S, Viikari-Juntura E, Alasalmi J, Busk H, Kauhanen A, Leinonen T, Solovieva S, Valkonen T (2020). Työkyvyttömyyteen ja osatyökyvyttömyyteen liittyvät etuudet ja järjestelmät [Social security benefits and schemes relating to work disability and partial work disability. Työpolitiikka ja työllisyysaste:tutkimukseen perustuvia johtopäätöksiä. Valtioneuvoston selvitys- ja tutkimustoiminnan julkaisusarja 2020:33 [Labour policy and employment rate:conclusions based on research. Publications of the Government's analysis, assessment and research activities;in Finnish, abstract in English]. Helsinki.

[3] Kausto J, Miranda H, Martimo KP, Viikari-Juntura E (2008). Partial sick leave--review of its use, effects and feasibility in the Nordic countries. Scand J Work Environ Health.

[4] Leoni T (2020). Graded work, the activation of sick-listed workers and employer participation in continental Europe. Soc Policy Soc.

[5] Kela [Internet Sickness allowances] https://www.kela.fi/web/en/sickness-allowances.

[6] Markussen S, Mykletun A, Røed K (2012). The case for presenteeism —evidence from Norway's sickness insurance program. J Public Econ.

[7] Kausto J, Viikari-Juntura E, Virta LJ, Gould R, Koskinen A, Solovieva S (2014). Effectiveness of new legislation on partial sickness benefit on work participation:a quasi-experiment in Finland. BMJ Open.

[8] Bethge M (2016). Effects of graded return-to-work:a propensity-score-matched analysis. Scand J Work Environ Health.

[9] Viikari-Juntura E, Virta LJ, Kausto J, Autti-Rämö I, Martimo KP, Laaksonen M (2017). Legislative change enabling use of early part-time sick leave enhanced return to work and work participation in Finland. Scand J Work Environ Health.

[10] Hernæs Ø (2018). Activation against absenteeism - Evidence from a sickness insurance reform in Norway. J Health Econ.

[11] Maas ET, Koehoorn M, McLeod CB (2021). Does gradually returning to work improve time to sustainable work after a work-acquired musculoskeletal disorder in British Columbia, Canada?A matched cohort effectiveness study. Occup Environ Med.

[12] Høgelund J, Holm A, Eplov LF (2012). The effect of part-time sick leave for employees with mental disorders. J Ment Health Policy Econ.

[13] Bosman LC, Twisk JW, Geraedts AS, Heymans MW (2020). Effect of partial sick leave on sick leave duration in employees with musculoskeletal disorders. J Occup Rehabil.

[14] Kausto J, Solovieva S, Virta LJ, Viikari-Juntura E (2012). Partial sick leave associated with disability pension:propensity score approach in a register-based cohort study. BMJ Open.

[15] Polvinen A, Laaksonen M, Rantala J, Hietaniemi M, Kannisto J, Kuivalainen S (2018). Working while on a disability pension in Finland:association of diagnosis and financial factors to employment. Scand J Public Health.

[16] Solovieva S, Pehkonen I, Kausto J, Miranda H, Shiri R, Kauppinen T (2012). Development and validation of a job exposure matrix for physical risk factors in low back pain. PLoS One.

[17] Solovieva S, Pensola T, Kausto J, Shiri R, Heliövaara M, Burdorf A (2014). Evaluation of the validity of job exposure matrix for psychosocial factors at work. PLoS One.

[18] Austin PC (2008). A critical appraisal of propensity-score matching in the medical literature between 1996 and 2003. Stat Med.

[19] Weitzen S, Lapane KL, Toledano AY, Hume AL, Mor V (2004). Principles for modeling propensity scores in medical research:a systematic literature review. Pharmacoepidemiol Drug Saf.

[20] Sullivan DF (1971). A single index of mortality and morbidity. HSMHA Health Rep.

[21] Jagger C, Cox B, Le Roy S (2006). and the EHEMU team Health expectancy calculation by the Sullivan method:a practical guide Third Edition. EHEMU Technical Report.

[22] van der Noordt M, IJzelenberg H, Droomers M, Proper KI (2014). Health effects of employment:a systematic review of prospective studies. Occup Environ Med.

[23] Hergenrather K, Zeglin R, McGuire-Kuletz M, Rhodes S (2015). Employment as a Social Determinant of Health:A Systematic Review of Longitudinal Studies Exploring the Relationship Between Employment Status and Physical Health. Rehabil Res Policy Educ.

[24] Waddell G, Burton AK (2006). Is work good for your health and well-being?.

[25] Shiri R, Kausto J, Martimo KP, Kaila-Kangas L, Takala EP, Viikari-Juntura E (2013). Health-related effects of early part-time sick leave due to musculoskeletal disorders:a randomized controlled trial. Scand J Work Environ Health.

[26] Streibelt M, Bürger W, Nieuwenhuijsen K, Bethge M (2018). Effectiveness of graded return to work after multimodal rehabilitation in patients with mental disorders:A propensity score analysis. J Occup Rehabil.

[27] Andrén D (2014). Does part-time sick leave help individuals with mental disorders recover lost work capacity?J Occup Rehabil.

[28] Schneider U, Linder R, Verheyen F (2016). Long-term sick leave and the impact of a graded return-to-work program:evidence from Germany. Eur J Health Econ.

[29] Kela 2021 [Internet] Statistical Yearbook of the Social Insurance Institution 2020.

[30] Caliendo M, Mahlstedt R, Mitnik O (2017). Unobservable, but unimportant?The relevance of usually unobserved variables for the evaluation of labor market policies. Labour Econ.

