# Supplementary material for: Working life expectancy and working years lost among users of part- and full-time sickness absence in Finland
Source: Scand J Work Environ Health. 2022 Dec 30;49(1):23–32. doi: 10.5271/sjweh.4054 (PMC10549914; doi:10.5271/sjweh.4054)
Supplement: Supplementary material [file SJWEH-49-23-S001.pdf]

Working life expectancy and working years lost among users of part- and full-time sickness absence in Finland [1]

by Elli Hartikainen, MSc,[2] Svetlana Solovieva, PhD, Eira Viikari-Juntura, MD, PhD, Taina Leinonen, PhD

1. Supplementary material
2. Correspondence to: Elli Hartikainen, The Finnish Institute of Occupational Health, P.O. Box 40, 00032 TYÖTERVEYSLAITOS, Finland [E-mail: elli.hartikainen@ttl.fi].

Supplementary table S1. Working life expectancy (WLE) and working years lost (WYL) among the matched part-time (pSA) and full-time (fSA) sickness absence groups at age 30 by gender, excluding multiple records of individuals

		**Men**					
		**pSA group**	**95% CI**	**fSA group**	**95% CI**	**Diff.**	**95% CI**
	**WLE**	20.50	19.81–21.19	17.93	17.26–18.59	*2.57*	1.22–3.93
**WYL**	**Partial work disability**	1.57	1.29–1.86	0.63	0.45–0.82	*0.94*	0.47–1.41
**WYL**	**Time-restricted work disability**	3.92	3.45–4.39	4.37	3.89–4.85	*-0.45*	-1.40–0.50
**WYL**	**Unemployment**	2.10	1.75–2.45	3.28	2.85–3.71	*-1.18*	-1.96– -0.40
**WYL**	**Other non-employment**	0.86	0.64–1.09	1.16	0.90–1.42	*-0.30*	-0.78–0.19
**WYL**	**Disability retirement**	0.88	0.68–1.09	1.96	1.67–2.25	*-1.08*	-1.57– -0.58
**WYL**	**Other permanent retirement**	1.93	1.53–2.33	2.44	2.17–2.71	*-0.51*	-1.18–0.16
		**Women**					
		**pSA group**	**95% CI**	**fSA group**	**95% CI**	**Diff.**	**95% CI**
	**WLE**	21.47	21.08–21.86	19.47	19.09–19.85	*2.00*	1.23–2.77
**WYL**	**Partial work disability**	1.94	1.76–2.12	0.96	0.84–1.09	*0.98*	0.67–1.28
**WYL**	**Time-restricted work disability**	4.34	4.06–4.62	4.58	4.30–4.86	*-0.24*	-0.80–0.32
**WYL**	**Unemployment**	1.47	1.30–1.65	2.68	2.45–2.91	*-1.21*	-1.61– -0.80

	**Other non-employment**	0.74	0.60–0.87	1.07	0.92–1.23	*-0.33*	-0.63– -0.05
	**Disability retirement**	0.51	0.42–0.61	1.41	1.28–1.55	*-0.90*	-1.13– -0.67
	**Other permanent retirement**	1.94	1.73–2.16	2.24	2.10–2.38	*-0.30*	-0.65–0.06

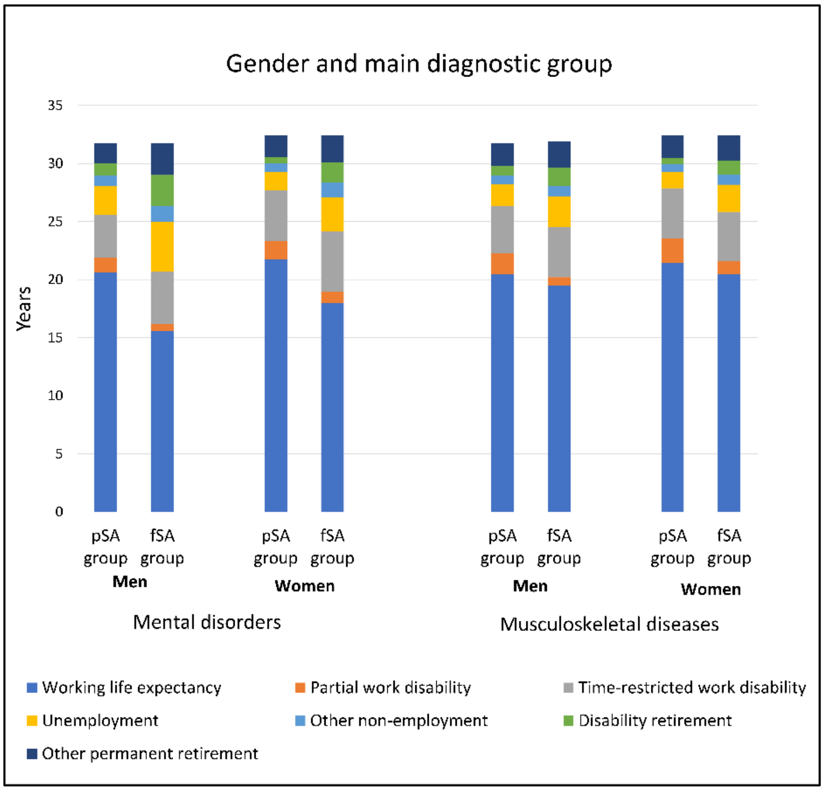

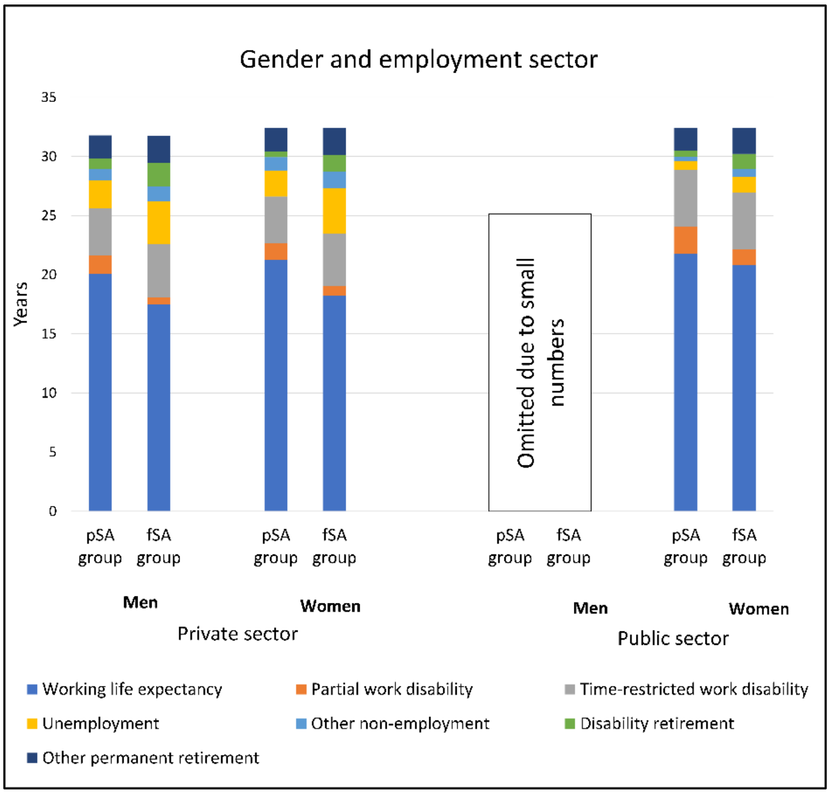

Supplementary figure S1. Working life expectancy and working years lost among matched part-time (pSA) and full-time (fSA) sickness absence groups at age 30 by gender, main diagnostic group and employment sector
